# “Living in Confinement, Stopped in Time”: Migrant Social Vulnerability, Coping and Health during the COVID-19 Pandemic Lockdown in France

**DOI:** 10.3390/ijerph191610084

**Published:** 2022-08-15

**Authors:** Maria De Jesus, Zoubida Moumni, Zara Hassan Sougui, Neeharika Biswas, Raquel Kubicz, Lionel Pourtau

**Affiliations:** 1 Center on Health, Risk, and Society, School of International Service, American University, Washington, DC 20016, USA; 2 Collegium de Lyon, Université de Lyon, 69002 Lyon, France; 3 Psychologie de la Santé, Université Lumière Lyon 2, 69365 Lyon, France; 4 Santé Publique, Université Claude Bernard Lyon 1, 69100 Villeurbanne, France; 5 Habitat et Humanisme, 69300 Caluire et Cuire, France; 6 Pôle Recherche & Innovation, Université Paul Valéry Montpellier 3, 34090 Montpellier, France

**Keywords:** COVID-19, lockdown, pandemic, migrants, asylum seekers, crisis, social vulnerability, health inequities, France

## Abstract

The COVID-19 pandemic has exposed health and social inequities among migrant populations. Less empirical evidence exists about the impact of COVID-19 lockdown measures on migrants. This study aimed to investigate the impact of the first lockdown in France between March and May 2020 on migrants’ lives and livelihoods. We adopted a social vulnerability framework to conceptualize how the pandemic and the consequential lockdown in France contributed to a ‘compounded crisis’ for asylum seekers and undocumented migrants. This crisis encompassed health, protection, and socio-economic challenges for migrants and exposed the shortcomings of existing government policies that exclude migrants and do not address the root causes of health inequities. The study draws on in-depth qualitative interviews conducted with 75 asylum seekers and undocumented migrants during the pandemic lockdown in the French regions of Auvergne-Rhône-Alpes and Île-de-France. The findings of this paper highlight the importance of implementing a cohesive pandemic response approach that views health as a fundamental inclusive right for *all* human beings and *all* policies as health policies to promote well-being for all.

## 1. Introduction

The coronavirus disease 2019 (COVID-19) pandemic led to governments in 157 countries introducing lockdowns or re-strictions to people’s movement and access to health and welfare support services as well as other rules including social distancing, use of masks, and quarantine. The French government introduced its first mandatory national lockdown on 17 March 2020 due to elevated cases and death rates of COVID-19 in the country [1]. This public health measure required the general population to stay at home except those carrying out an essential job (referred to as a ‘key worker’ in the domains of transportation, education, food, and health), to buy necessary items, or to engage in physical activity.

Evidence demonstrates that the pandemic disproportionately affected socially vul-nerable populations, including migrants [2,3,4,5]. The pandemic exposed and exacerbated health and social inequities among migrant and ethnic/racial groups [6]. Less is known about the firsthand impact of the COVID-19 lockdowns, specifically on migrant populations in France. One recent French qualitative study in Lyon and Paris on homeless individuals and migrants (undocumented, asylum seekers, or refugees) in shelters revealed the negative impact of the first lockdown on these populations [2]. Similarly, another qualitative article on migrants in France described the negative effects of confinement related to the COVID-19 pandemic on French-speaking Sub-Saharan undocumented migrants [3]. Similarly, our study contributes to this understudied area of research by exploring the impact of the first lockdown in France on migrants’ lives and livelihoods from their perspectives. This information is critical to inform future preparedness response plans that include migrants so as not to further exacerbate vulnerabilities among these populations.

### 1.1. Defining Migrant Terminology Used in the Study

While the term refugee is precisely defined in the 1951 Convention relating to the Status of Refugees and the 1967 Protocol 3, migrants are a heterogeneous group with no international consensus [7]. The umbrella term ‘migrant’ is defined as a person who moves away from his or her place of usual residence, whether within a country or across an international border, temporarily or permanently, and for a variety of reasons [8]. Table 1 describes a common typology of migrants. For the purposes of this study, we adopt the term “migrant” which includes the labels in Table 1.

Their asylum case at the host country with the aim of obtaining refugee status, they may also be documented or ‘undocumented’ (“*sans papiers*” in French). There is also no universally accepted definition of ‘irregular’ migration. The International Organization for Migration (IOM) defines it as “movement that takes place outside the regulatory norms of the sending, transit, and receiving country” [8]. From the perspective of destination countries, it refers to entry, stay, or work in a country without the necessary authorization or documents required under immigration regulations. Migrants escaping conflict and persecution in their countries and seeking protection in another country may be counted as undocumented migrants at the moment of crossing the border, but their status may change once they apply for asylum [9,10] or are regularized.

### 1.2. Social Vulnerability: A Conceptual Lens to Understand the Experiences of Migrants

The concept of social vulnerability is used across a variety of fields and disciplines, including global health, development, disaster management, economics, sociology, anthropology, geography, and environmental studies. The concept emerged within the natural hazards and disaster management literature, emphasizing the exposure risk and the amount of damage caused by a particular hazard from a technical or engineering sciences perspective [11,12]. Rather than simply examining risk exposures, social scientists transformed the concept to consider the vulnerability that pre-exists in a system before it encounters a hazard.

The negative impact of a pandemic on migrant health and well-being are exacerbated by antecedent social determinants, including poverty, discrimination, social exclusion, low levels of education, poor public infrastructure, and uneven distribution of resources that amplify social vulnerability [5,11,13]. From this perspective, the COVID-19 pandemic is conceptualized as a crisis or disruptive event that occurs at a rate and magnitude that exceeds normal capacity (e.g., increasing cases, hospitalization, and death rates of COVID-19).

**Figure 1:**
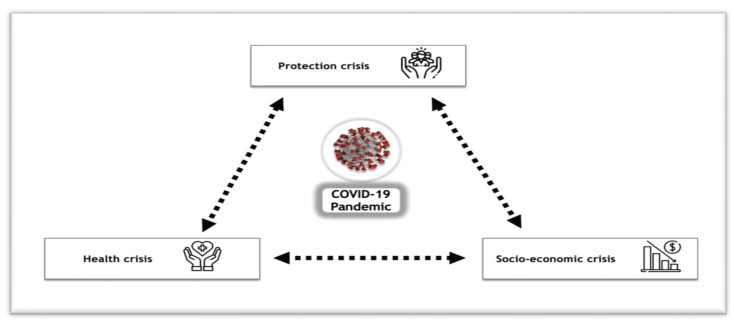
Social vulnerability: a conceptual framework to interpret the impact of the COVID-19 pandemic and subsequent lockdown on migrants (adapted from UN Sustainable Development Group) (Reprinted/adapted with permission from ref. [15]. Copyright 2021, UN Sustainable Development Group).

In this article, we adopt the lens of social vulnerability as a conceptual framework to help interpret the impact of the pandemic and consequential lockdown. The notion in this framework is that a ‘compounded crisis’, which encompasses a health crisis, a protection crisis, and a socio-economic crisis [14], arises from existing vulnerabilities reinforcing each other and is amplified during a pandemic and subsequent lockdown, creating a detrimental cycle (Figure 1).

### 1.3. A Health Crisis

Data from the World Health Organization (2020) demonstrated that 93% of countries experienced disruption in one or more of their services for mental, neurological, and sub-stance use disorders [16]. Mental health services were cut during the pandemic, given the insufficient number of health workers, the lack of personal protective equipment (PPE), and the conversion of mental health facilities to quarantine/treatment facilities [17]. The pandemic, therefore, led to worsened pre-existing mental health outcomes or to new con-ditions among migrants [17,18,19,20,21].

Apart from the U.K, Norway, Sweden, Denmark, Finland, and Iceland, European countries did not report COVID-19 statistics according to migrant status or ethnicity [22]. Scandinavian countries reported higher rates of COVID-19 cases, mortality, and morbidity among migrant populations [23]. For example, in Sweden, in comparison to Swedish-born individuals, migrants from Somalia faced a ninefold excess risk in mortality, sixfold for migrants from Lebanon, fivefold for migrants from Syria, and a two-to-threefold for migrants from Turkey, Iran, and Iraq [23]. In Denmark, migrants of non-Western origins comprised 15% of COVID-19 hospital admissions despite constituting 9% of the total population [23]. In France, population-wide data on morbidity and healthcare are not col-lected according to ethnic and migrant status [24]. However, data on all-cause mortality rates revealed that persons who are foreign-born had, on average, double the rates of all-cause mortality (some of which were due to COVID-19) compared with the native-French population between March and April 2020, when the virus was surging [25]. Excess COVID-19-related mortality (i.e., a 118% increase compared with the preceding year) was also observed in Seine-Saint-Denis (located in the North of Paris), the poorest district in France with the highest immigrant population (30% in comparison to France’s at 10%) [26,27].

Furthermore, the stringent measures towards migrants regarding healthcare provision in France have made it more difficult for migrants to seek healthcare. The 2019 *L’aide médicale de l’État* (AME), or Federal Medical Assistance reform, meant that asylum seekers must complete a 3-month “délai de carence” (waiting period) in France prior to accessing basic healthcare coverage. New restrictions on the type of care available were also introduced, for example, excluding psychiatric support [28].

### 1.4. A Protection Crisis

Most governments instituted measures such as social distancing, lockdown proce-dures, and mask mandates in response to COVID-19 case and death rates at the time [29,30,31]. Implementing government-mandated COVID-19 precaution measures proved particularly challenging for migrants residing in precarious living conditions, which con-tributed to increased vulnerabilities and a protection crisis, and in turn, elevated risk for COVID-19 infection and death among this population, as described above.

Unsanitary and overcrowded living conditions were already an existing issue prior to the COVID-19 pandemic and were exacerbated during the lockdown and the pandemic. For example, the Moria camp in Lesbos, Greece dubbed as “Moria jungle,” housed 17,000 migrants above capacity and eventually burnt down in September 2020, leaving over 20,000 residents without shelter [32]. Perilous conditions were not restricted to migrant camps in the Mediterranean; many urban and perceived ‘progressive’ states have fostered the same adverse living conditions. In November 2020, the French police destroyed a mi-grant camp at the Place de la République in central Paris [33]. The substandard living conditions in the French capital saw migrants with little to no access to sanitation facili-ties and reliant on food donation points by local organizations and civil society [33]. Over 85% of respondents slept in tents, under bridges, or on damp mattresses on the floor [33]. Between 2015 and 2017, the unhygienic and overcrowded conditions combined with a lack of essential resources (e.g., hygiene products) led to recurring outbreaks of scabies [34]. Overall, these adverse physical environmental conditions further augment the popu-lation’s vulnerability to disease outbreaks. These living conditions were worsened during the COVID-19 lockdown and the pandemic.

### 1.5. A Socioeconomic Crisis

Migrants face major hurdles in economic integration in France, given that they have little to no access to the labor market [35]. Undocumented migrants face *de jure* work restrictions (i.e., not legally permitted to work given that they are not afforded the same labor rights in law as citizens) and de facto struggles when working illegally [35]. Most migrants who work are limited to the informal sector with no protections and, thus, higher vulnerabilities.

Evidence highlights the economic and labor challenges faced by migrants due to the COVID-19 pandemic, including increased difficulty in accessing informal labor market participation [29]. The International Labour Organization (ILO) found that these informal sectors were highly impacted by the pandemic, leading to the widespread loss of livelihood and the resultant poverty increase among migrants in Europe [36]. The loss of jobs and informal income-generating opportunities, therefore, led to even deeper financial and food insecurity for migrants than before the pandemic [33].

## 2. Methods

This study was part of a larger mixed methods research project in collaboration with a community partner, *Habitat et Humanisme*. In 2016, *Habitat et Humanisme* expanded its mission to aid asylum seekers and refugees in France. Its three main aims are (1) to provide accommodation centers for asylum seekers and refugees where they can obtain social, administrative, legal, and integration support; (2) to develop integration programs through training, employment, housing, and culture for refugees; and (3) to conduct research on migrants and their experiences in the host country. Approval for this study was obtained from the Institutional Review Board of the Université de Lyon.

Between September 2019 and February 2020, we began our field research with asylum seekers and refugees. We contacted 16 centers that served migrants in the southeast-central region of Auvergne-Rhône-Alpes and in the north-central region of Île-de-France. Twelve of the centers were affiliated with *Habitat et Humanisme*. Our partnership facilitated our entry into the centers. The first three authors went to each of the centers and held study information sessions to describe the study and the eligibility criteria (at least 18 years of age; migrant status; and currently residing in France) and answer any questions from potential participants. We also emphasized that participation was voluntary. Following the information session, interested participants enrolled in the study. Data collection for the larger research project consisted of ethnographic observations, in-depth, semi-structured interviews, and a survey.

Our field data collection was halted abruptly due to the COVID-19 pandemic. On 16 March 2020, President Macron announced a mandatory home lockdown for 15 days starting at noon on 17 March 2020. This was extended twice and ended on 11 May 2020. Given the pandemic and lockdown situation, we decided to expand our research and developed a semi-structured qualitative interview with the aim of gaining an in-depth understanding of the real-time impact of the pandemic and lockdown from the perspectives of migrants who were either asylum seekers or undocumented. Six months of fieldwork on the larger research project with migrants allowed for much more rapid access to conduct this follow-up research than would normally be the case for community-based qualitative research.

We employed a qualitative, inductive research design, which is concerned with an emic, idiographic approach to research [37]. This approach is focused on the experiences of specific participants (i.e., migrants) and the unique richness of a phenomenon (i.e., their perspectives and experiences living through a pandemic and lockdown). This was the design of choice given the lack of information about this research topic. A semi-structured interview guide was developed to ensure consistency across interviews. Participants were asked open-ended questions with probing questions.

This article presents data from the hour-long phone interviews we conducted with 75 migrants during the pandemic lockdown between March and May 2020. Approximately 120 asylum seekers were contacted, and the purpose of the study were clarified; we were not able to reach about one-third, and several refused to participate in the study. We first collected demographic information from those participants who agreed to participate in the study and then scheduled the interviews. Due to sensitivity regarding obtaining written consent from a migrant population who was seeking asylum and/or undocumented, we obtained informed oral consent instead from each participant prior to the interview. The interviews were conducted in English or French, and an interpreter was used for the participants who stated that they preferred to do the interview in their native language. Each interview was recorded and transcribed into English. Data saturation, that is, when more interviews were not adding any new themes or concepts to what was already known, was achieved [38].

### Data Analysis

Qualitative thematic analysis was used to analyze the interview data [39]. Based on Corbin and Strauss’ qualitative analytic methods, this analytic technique is grounded in the process of coding similar responses from the sample and using those codes to discern important themes and patterns across participants [39]. The first three authors followed an iterative process of independently reading the interview transcripts, developing a codebook, applying a priori codes, and comparing and revising codes using ATLAS.ti (version 8.0, Berlin, Germany). Using a line-by-line coding method, we each added emergent codes from the data and modified the codebook as needed during the analytic process. Subsequently, we located patterns in the codes and collapsed codes into themes. This was an interpretive process where we searched for meanings at the level of coding as well as contextualized understandings within the data at the level of interpreting findings [39].

During weekly online debrief meetings, we discussed points of disagreement. Stability and agreement are the most relevant types of reliability for textual data. After revisions, we achieved an interrater reliability of 95 percent. To see if this agreement was due to chance, the intercoder reliability was tested using Cohen’s Kappa [40]. The overall Kappa coefficient was 0.96. To further ensure data validity, the lead author conducted a member check with a smaller subgroup of migrants (*n* = 5) who did not participate in the study but possessed similar sociodemographic characteristics as the study participants. Consensus was reached on key themes related to the perceived impact of the lockdown.

## 3. Results

Table 2 summarizes the sociodemographic characteristics of the study sample. Most participants were male (85%), and the median age for the sample was 26 years old. Approximately 75% of the sample had a primary or secondary level education. While almost 62% of participants had an intermediate level of French language comprehension, more than half (56%) had only a beginner level of oral expression in French. Slightly more than half of the participants were from an African country (55%), while 45% were from Afghanistan. The sociodemographic data for this study is comparable to similar studies with migrants in France [2,3].

The salient themes reflecting the impact of the pandemic and lockdown from the perspectives of the migrants themselves are reported below. Pseudonyms are used for all the participants. The findings below depict how the pandemic and subsequent lockdown contributed to a ‘compounded crisis’ (i.e., a health, a protection, and a socioeconomic crisis). A social vulnerability lens elucidates how the vulnerabilities experienced by the migrants during the lockdown reinforce and amplify each other.

### 3.1. Challenges in Following the Public Health Guidelines for Stopping the COVID-19 Spread

Although most of the migrants stated their desire to follow public health guidelines, they recounted not being able to easily follow the guidelines of social distancing and handwashing. Moussa, a 23-year-old male migrant from Guinea, expressed the challenges posed by his living conditions: *“It is practically impossible to follow the social distancing rule. We live in a small physical space and it is overcrowded*”. Another migrant from Nigeria, 25 years of age, spoke about informational and resource barriers to implementing the COVID-19 public health measures: *“Some of the guys are not wearing masks. There is a lack of resources and information. Some are not wearing it correctly. They are placing it underneath their nose or letting it hang by one ear. We also do not always follow the hand sanitization rule. Sometimes we are missing hand soap or hand sanitizer here*”. In addition to a protection crisis, the migrants also experienced challenges related to food and financial insecurity.

### 3.2. Food and Financial Insecurity

Approximately three-quarters of the migrants spoke of a disruption in their food intake during the lockdown because of lack of money and other resources, which was ex-acerbated due to the lockdown and the pandemic. Mariame, a 28-year-old female from Guinea, recounted her experience: “*During the lockdown, I do not have enough money to buy enough food for the week. To get to those stores that sell food more cheaply, I need a tram ticket, and I do not have money for that. There are days when I go hungry*”. *Other migrants expressed feeling stressed and worried due to their financial situation during the lockdown.* Abdul, a 27-year-old male from Afghanistan stated: “*With the lockdown I am very stressed thinking that I do not have enough money for myself nor to send to my wife and kids back home*”. The pandemic and lockdown also negatively affected migrants’ social environment, and, in turn, took a toll on their physical and mental health, as described below.

### 3.3. Formal Social Networks Disrupted

During the lockdown, most expressed that they felt cut off from their more formal social networks comprised of center staff, social workers, and volunteers. These formal networks were disrupted during the lockdown because there were fewer staff members at the migrant reception centers during that period. Without direct support from staff, the migrants also experienced barriers to scheduling needed administrative, medical, and social service appointments. For many of the migrants, the staff members were their main source of connection to a French person. The disruption of these relationships contributed to feelings of loneliness and social isolation among many of the migrants. Oumou, a 22-year-old male migrant from Mali recounted his experience during the lockdown period:

*I am completely lost. The people I depend on at the center are not here. Days pass, and I cannot get anything done. I can’t schedule my asylum application appointment. Everything is shut down. I need a signature from the center staff on one of the forms and they are not here. There is nothing to do but wait. I am getting more anxious as the days go on. It seems like it is never-ending, and I feel very alone and desperate without the staff here.*

A few migrants described how they were able to obtain some staff phone support, but it was very limited, which made it difficult for them to adapt to this unprecedented situation.

Most of the asylum seekers also described not having all the necessary information related to COVID-19 and the lockdown and described feeling abandoned by the staff members. Abdul-Ali, a 26-year-old male Afghan migrant reported: “*It is like they (staff at center) left us all alone to fend for ourselves with minimal resources. We need the support from staff in such a difficult situation. There is a lot of confusion and a lack of information during the lockdown. I am not sure what to do or where to go. There is no one here. I do not speak French and do not have any papers or other resources…*” In addition to disrupted formal social networks, all the participants mentioned how the pandemic and lockdown negatively affected their daily and weekly routines.

### 3.4. Daily and Weekly Routines Disrupted

The lockdown affected the migrants’ routines. All the asylum seekers would typically attend French as a second language courses twice a week with an instructor who was a native speaker. For these asylum seekers, the instructor was a consistent source of contact with the French language and culture, and therefore, no longer having this connection made them feel even more disconnected from French society. Some participants shared that their instructor had tried to continue the course via WhatsApp, but this proved to be too challenging and was discontinued. Volunteers were also not able to come to the centers where migrants resided during the lockdown. A 26-year-old male Afghan asylum seeker stated:

*I look forward to seeing the college students who come every week to the center. We play games, exchange, and have tea together. I learn more about French culture and the language by talking with the volunteers. I miss doing things with them. I spend my time doing nothing all day. The volunteers are all gone. When will I get to see and talk with them again?*

A few migrants who were more proficient in French also volunteered in different organizations. These opportunities had ceased abruptly and indefinitely due to the pandemic. These disruptions meant less interaction and support from their social networks. As a 23-year-old female migrant from Mali stated: “*Volunteering gave me a good excuse to get out and make myself useful. I also made new friends*”. Being cut off from their social networks and breaking with their everyday routines and activities contributed to a health crisis, including experiences of social isolation and loneliness.

### 3.5. Social Isolation and Loneliness

All the participants stated that their social isolation intensified during the lockdown. During this period, the French government had decreed that people should only leave their homes for essential activities, for example, to shop for food or other necessities or to exercise, and that they should limit their distance to one kilometer (0.62 miles) from their home. A Somalian 20-year-old male, Abdirahim, stated, “*I feel confined in my room. There is no one around me. I feel very lonely. Without papers, I feel even more confined as I am not a full member of this society*”.

Similarly, another 20-year-old male asylum seeker from the Ivory Coast, Kouakou, described his experience this way: “I am living in confinement, stopped in time. I could not really go anywhere or see anyone other than the people who live with me in the container. I feel isolated from the rest of the world”. Although they could physically go outside during the lockdown period, slightly more than half of the participants had no interest in doing so, stating that they felt “depressed,” “stressed”, or “bored”.

Most of the migrants also shared that they had lost interest in eating, personal hygiene, or keeping their living space tidy. All the participants also shared that during the lockdown they experienced poor sleep quality, either “*not sleeping well at all*” or “*too little*” or the other extreme of “*sleeping all day*” or “*most of the day*”. In addition to feeling socially isolated and lonely, all the participants reported that they felt an added layer of worry and stress with being away from their families during the pandemic.

### 3.6. Intense Worry for Themselves and Their Distant Family Members

During the lockdown, most of the participants expressed that they felt even more vulnerable given that they were worried and stressed about the pandemic for themselves and their distant family members. As Seydou, a Malian 26-year-old male asylum seeker, relayed: “*If one of us gets sick, what will happen to us? Where will we go? Where will they put us? Who will help us? Many of us are here alone with no one to help us if we get sick. We do not have legal papers, which will make it more difficult*”.

All the migrants also shared concerns about the health and well-being of their distant family members. These family members were in countries with less resources, an inadequate health infrastructure, and where there was a lack of information or misinformation about COVID-19. As Djoëlle, a 24-year-old female asylum seeker from the Democratic Republic of the Congo explained: “*I miss my kids and family back home and I am very worried for their well-being. COVID-19 is scary and there is nothing and nobody there to help them. They do not even have access to the right information. The healthcare system is bad there. What will they do if they get sick?*” Most of the migrants also felt excluded from mainstream society during the lockdown.

### 3.7. Social Exclusion, Marginalization, and Fear of Deportation

During the lockdown, migrants experienced intensified social exclusion, marginalization, and fear of deportation. Nawaskhan, a 25-year-old male migrant from Afghanistan stated, “*We are not French. We do not speak the language well. As migrants, what rights do we have to complain? We are here, yet we are excluded. During this stay-at-home time, we cannot do anything. Where do we go for help if we need it? We have not been granted refugee status, so we are marginalized*”.

Many asylum seekers shared that they felt “*uncomfortable*” or “*fearful*” leaving their room, apartment, center, or container where they lived. Given their undocumented status, many of them did not feel comfortable completing a paper copy or online version of the “Attestation de déplacement” (i.e., government-issued travel certificate), which was required every time one left one’s home during the lockdown period to “prove” their purpose for leaving home. Genet, a 28-year-old female migrant from Eritrea, stated, “*I do not have my French papers, so I do not feel at ease filling out an ‘Attestation’ (travel certificate)*”. Similarly, Mohamed, a 27-year-old male Congolese asylum seeker’s narrative revealed his fear of being deported:

*It is too risky to go outside, especially because I do not have my resident permit or any papers. Without my papers, I am excluded. Now they are stopping people and asking them for their attestation de déplacement (travel document). I also do not want to be stopped by the police and risk paying a fine of 135 euros for not having my attestation. I am afraid of going outside. I could get stopped and deported.*

The asylum seekers were already in a “limbo” situation having to wait a longtime (in some cases, several years) to hear news about the outcome of their asylum case and whether they could remain in France with legal status. Along with the lockdown in place, this produced even stronger feelings of uncertainty among migrants regarding their future.

### 3.8. Uncertain Futures

Due to the lockdown, the asylum administrative processes had all been halted given that government offices had been temporarily closed until further government notice. As a result, all the participants expressed feeling greater uncertainty about their future and the outcome of their asylum case. In the words of Bahiri, a 24-year-old male Afghan asylum seeker: “*It is even harder now during the lockdown to work towards future goals. I am just waiting*”. Tabish, also a 24-year-old male migrant from Afghanistan, explained: “*When will my asylum application be processed? I have been waiting for 13 months already and now with the lockdown, it will take even longer. I feel even less confident about my future*”. Four participants who already had court appointments for their asylum proceedings received news of their appointments being postponed indefinitely.

Not being able to plan for their futures took a heavy toll on the migrants’ mental and physical health and well-being. Many of them stated that they were experiencing headaches, backaches, anxiety, depression, and suicidal thoughts. Nine participants also mentioned that they could not go to the doctor due to structural barriers. Abdo, a 23-year-old male Sudanese migrant, explained: “*I was waiting and waiting for my French healthcare insurance card. Before the pandemic, there was a delay already; you had to wait at least three months. But with the pandemic and the lockdown now, I do not know when I will get it. If I get sick, I do not know what will happen to me*”. Despite all the challenges they were facing during the lockdown, the migrants demonstrated different forms of coping too. They turned to each other for different types of support when they needed it most.

### 3.9. Migrant Solidarity and Reliance on Informal Support Networks

About a third of the asylum seekers mentioned that there was a strong sense of solidarity and social support among fellow asylum seekers during the lockdown. Aboubacar, a 29-year-old male migrant from Guinea stated: “*We decided to form an informal sport group during the lockdown. We use the space outside the container to work out together. It is a great way to feel less lonely and get energy for ourselves and from each other*”.

Kamal, a 20-year-old male Sudanese asylum seeker, expressed relying on these informal networks for tangible support during the lockdown: “*We depend on one another right now. When I need money, my friends at the center help me out and give me some money. They know I am more desperate, and they help me out during this hard time during the lockdown*”. Most of the participants also recounted being able to depend on fellow asylum seekers for emotional support. Babacar, a 26-year-old male migrant from Senegal, explains: “*This is tough right now with the confinement. I am down. I have not been out of my room in three weeks. This is the first time today that I went to the salon to talk to the other guys there. When I am down, I isolate myself in my room. They help me feel better. We listen to music and watch some YouTube videos together. They help me feel a little better*”. There were many other examples of solidarity among migrants during the lockdown period. They also revealed different forms of coping.

### 3.10. Forms of Coping

Besides mutual social support, most migrants found other ways to cope with the negative consequences of the lockdown. Many of them spoke about needing to stay strong for themselves and their families, including those relatives with whom they stayed in touch via social apps on their phones, such as WhatsApp. Seydou, a 24-year-old male migrant from the Ivory Coast, expressed: “*What choice do I have? I need to be there for my wife and three children in the country. I need to put a smile on their faces. I talk to them three times a week. I do not burden them with my worries. I tell them everything is ok, even though we are having a lockdown here*”.

Omran, a 28-yer-old male asylum seeker from Afghanistan, shared how the lockdown situation was beyond his control and how he used his spiritual faith as a coping mechanism: “The confinement is not something I can control. We must make the best out of this situation. *In sha’Allah!* I leave it in Allah’s hands. He will help me, and all of us make it through this difficult time. I pray and ask Allah for strength”. Many of the Arabic-speaking migrants verbalized the common Arabic term “*In sha’Allah*”, an equivalent to the English expression “God willing” or “if God wills” when they were referring to “*getting through*” the lockdown. The term expresses the belief that nothing happens unless God wills it, and that God’s will supersedes all human will. Most of the participants demonstrated different forms of coping as they faced multiple challenges during the mandatory lockdown.

## 4. Discussion

The findings reveal that migrants experienced increased food and financial insecurity as well as disruptions of formal social networks and routines. These vulnerabilities led to more social isolation and loneliness, as well as more worry for themselves and for their relatives who were left behind in their countries of origin. During the lockdown, feelings of social exclusion, marginalization, and fear of deportation among migrants escalated. In addition, they worried about the consequences of the pandemic on both their immediate and future lives.

Analysis of the migrants’ narratives showed how these negative effects on their social, physical, and psychological well-being converged during the lockdown. Our study findings are consistent with similar studies conducted in France [2,3,41]. First, the gap in mental and physical healthcare for migrants only worsened during the lockdown as migrants were cut off from their regular social networks of volunteers and aid workers. The unmet needs of migrants piled onto the already large burden of being a migrant without documentation in France.

Second, the financial challenges that migrants were experiencing during the pandemic added another layer of vulnerability to their lives as stipends that they typically received from the government were either unreliable or stopped completely, especially as funds were being diverted to pandemic healthcare efforts [33,36]. Moreover, informal income-generating opportunities were now unavailable, contributing to the financial insecurity experienced by many migrants. This also led to food insecurity as many migrants who relied on aid organizations for food were left with little to no resources.

Third, there was a lack of security and protection for migrants, many of whom were living in precarious living conditions, making it nearly impossible to follow public health measures and putting them at an increased risk for contracting COVID-19. The health, socio-economic, and protection crises exacerbated by the COVID-19 pandemic and lockdown disproportionately affected migrants, given their severely limited ability to access healthcare, education, work, social protections, and other fundamental human rights even prior to the pandemic. Furthermore, the pandemic exposed the negative health and social outcomes among migrant groups that have resulted from long-standing structural inequities.

On a larger scale, the gaps left for migrants call into question policies for the general population. The global pandemic was a test for our policies and social protection measures, and we failed. The difficulties the migrants were experiencing during the pandemic served as a litmus test that exposes failures in policies and practices that do not address the social determinants of health, as evidenced by the negative outcomes for migrants, who are among the most vulnerable populations on the front line.

Specifically, in France we observe that a major barrier to address migrant health and well-being is the lack of data on migrants and their social conditions [24]. These practices and norms stem, in part, from an assimilationist view and practice of foreign-born individuals and ‘color-blindness’ ideology. The lack of data, including epidemiological data about migrants, poses multiple challenges for researchers and policymakers, such as the underestimation of the number of ‘*sans papiers*’ (i.e., undocumented) and the inability of researchers to document the full extent of the problem.

The COVID-19 response plan in place during the onset of the pandemic and lockdown was a policy that excluded migrants and therefore worsened their health and well-being. Migrant aid organizations have filled the gaps left by the state by providing migrants with as much care as they were able to provide. The vaccine rollout added another layer of complexity as the French government was hesitant to offer vaccinations to undocumented migrants [42]. Aid organizations once again stepped in to fill the gaps in healthcare left by the state, and in July 2021, *Médecins Sans Frontières* (Doctors Without Borders) began offering vaccines to migrants [41,42].

### 4.1. Study Limitations and Strengths

Limitations of this study include a small sample, which makes it difficult to know whether the findings are valid for the overall migrant population in France. This was the design of choice given the lack of information about this research topic and the difficulty in reaching this marginalized population. Despite the limitation, this study was innovative, and the use of a rigorous, qualitative methodology allowed us to generate in-depth knowledge from the perspectives of migrants living through the first pandemic lockdown in France. We were also able to achieve data saturation with the sample. Other study strengths include adopting a community-academic research partnership model, using multiple coders to check reliability, and conducting member-checks to ensure data validity.

### 4.2. Policy and Practice Implications

Study findings highlight the importance of understanding the negative effects of the COVID-19 pandemic and lockdown measures on migrants’ lives and livelihoods to develop effective policies and measures that can either prevent or mitigate these effects in a future pandemic. First and foremost, vulnerable populations such as migrants require special attention from policymakers in their response to a pandemic, given the migrants’ adverse living situations. Safe living conditions that ensure appropriate personal protective equipment and non-crowded living quarters are essential to prevent the exposure and spread of a virus among migrants and spread to others outside their living spaces. Moreover, inclusive policies that ensure equitable access to healthcare, including prevention and treatment, testing, and new therapeutics, are critical to protecting the whole population.

Second, the collection and dissemination of surveillance data and sociodemographic data including age, country of birth, migration status, and self-reported race/ethnicity will help determine the relative contribution of each of these driving factors for the observed health disparities and identify areas of unmet need. We must also advocate for the strengthening of public health datasets so that data can be collated and shared more effectively, as this is an essential step to guide policy, healthcare provision, prevention, and intervention efforts. These data systems will be important in both supporting the response to a future pandemic as well as new surges and in ensuring a long-term response to better tackle health inequities.

Third, public health efforts that provide messaging and interventions adapted to the linguistic, cultural, and social circumstances of migrants will be crucial to effectively prevent transmission within and beyond these communities.

Fourth, it is important to recognize the vital role that civil society organizations and other non-governmental actors play in providing different types of much-needed support to migrants during a pandemic. Their efforts highlight how the French government needs to adopt an intersectoral approach to mitigating the effects of a pandemic.

Fifth, the COVID-19 pandemic has shone a spotlight on health inequities and has created an opportunity to address the social determinants of health underlying these inequities. To date, policymakers have yet to adopt an approach that views *all* policies (including immigration policy) as health policies. This approach, referred to as the *Health in All Policies* approach, highlights how all sectors in the government, such as transportation, education, housing, and employment, have healthcare implications [43,44,45]. For example, during the COVID-19 pandemic, transportation policy is important because an individual who has COVID-19 who relies on public transportation for work can be exposed to the virus easily and then spread the virus to many people through close contact, which makes wearing masks, regular cleaning, and more frequent buses and trains a necessity to cut down on crowds and curb the spread of the virus.

In addition, implementing a *Health in All Policies* approach through the lens of migration during a global pandemic underscores the importance of achieving regional and global policy coherence. As we observed with the case of COVID-19, response policies varied by country, which made it difficult to curb the spread of COVID-19. The statement that “no one is safe until everyone is safe” highlights the need for a united global response to a pandemic [46]. Having regional and global coordination of response policies headed by health ministers across countries would ensure policy coherence across the board and a universal standard of practices that could potentially curb the global spread of a contagious disease like COVID-19.

Lastly, creating these coherent pandemic policies would also potentially help to depoliticize COVID-19 and healthcare for migrants. A case in point is Portugal. At the beginning of the pandemic, the Portuguese government implemented a policy that treated migrants within its borders as if they were residents, giving them full access to public services, including healthcare [47]. Portugal’s policy serves as an example of incorporating a multisectoral approach and depoliticizes the care of migrants under the state system. Future studies examining how policy changes that promote equity and inclusion influence health outcomes among migrants are recommended.

## 5. Conclusions

Health is a fundamental human right for *all* human beings despite income, ethnicity/race, gender, age, country of origin, and other factors, and is indispensable for the exercise of other human rights. Thus, the right to health is an inclusive right that extends not only to healthcare but also to the underlying determinants of health, such as adequate sanitation, sufficient and nutritious food, and safe living and working conditions. Inclusive, responsive, and coherent policies and practices that apply health and healthcare to *all* sectors and regardless of migration status, are essential. Ultimately, this is the way forward to minimizing the burden and transmission of infectious diseases and promoting health equity for all.

## Figures and Tables

**Table 1:** Defining common migrant terminology, adapted from International Organization for Migration (Reprinted/adapted with permission from ref. [8]. Copyright 2021, J. Redpath-Cross & R. Perruchoud).

**Asylum Seeker**	A person who seeks safety from persecution or serious harm in a country other than his or her own and awaits a decision on the application for refugee status under relevant international and national instruments.
**Economic Migrant**	A person leaving his or her habitual place of residence to settle outside his or her country of origin in order to improve his or her quality of life. This term is often loosely used to distinguish from refugees fleeing persecution and is also similarly used to refer to persons attempting to enter a country without legal permission and/or using asylum procedures without bona fide cause. It may equally be applied to persons leaving their country of origin for the purpose of employment.
**Irregular Migrant**	A person who, owing to unauthorized entry, breach of a condition of entry or the expiration of his or her visa, lacks legal status in a transit or host country. The definition covers, inter alia, those persons who have entered a transit or host country lawfully but have stayed for a longer period than authorized or subsequently taken up unauthorized employment (also called clandestine/undocumented migrant or migrant in an irregular situation). The term ‘irregular’ is preferable to ‘illegal’ because the latter carries a criminal connotation and is seen as denying migrants’ humanity.

This is a condensed list and is not exhaustive.

**Table 2:** Sociodemographic characteristics of study sample (*n =* 75).

Gender, *n* (%)	
Male	64 (85.33)
Female	11 (14.67)
Age (years), median	26
Education, *n* (%)	
Primary	22 (29.33)
Secondary	34 (45.33)
Post-secondary	17 (22.67)
Little or no schooling	2 (2.67)
Level of Comprehension in French, *n* (%)	
Beginner	19 (25.33)
Intermediate	46 (61.33)
Advanced	10 (13.34)
Level of Oral Expression in French, *n* (%)	
Beginner	42 (56.00)
Intermediate	28 (37.33)
Advanced	5 (6.67)
Country of Origin, *n* (%)	
Afghanistan	34 (45.33)
Guinea	7 (9.33)
Mali	6 (8.00)
Ivory Coast	5 (6.67)
Nigeria	4 (5.33)
Senegal	4 (5.33)
Sudan	3 (4.00)
Somalia	3 (4.00)
Eritrea	2 (2.67)
Congo	2 (2.67)
Chad	2 (2.67)
Madagascar	2 (2.67)
Pakistan	1 (1.33)

## Data Availability

Data can be made available upon reasonable request.
